# Animal models of trigeminal neuralgia: A commentary

**DOI:** 10.1177/1744806920980538

**Published:** 2020-12-27

**Authors:** Kaj Fried, Per T Hansson

**Affiliations:** 1 Department of Neuroscience, Karolinska Institutet, Stockholm, Sweden; 2 Department of Molecular Medicine and Surgery, Karolinska Institutet, Stockholm, Sweden; 3 Division of Emergencies and Critical Care, Department of Pain Management and Research & Norwegian National Advisory Unit on Neuropathic Pain, Oslo University Hospital, Oslo, Norway

Trigeminal neuralgia (TN), a classical neuropathic pain state, is a time-honored diagnosis and a well characterized condition with a variety of possible etiological backgrounds. It has recently been classified and described in detail by the International Headache Society (http://www.ihs-headache.org.) and in a consensus report by the European Academy of Neurology.^1^ Three diagnostic subgroups have been identified: classical, symptomatic and idiopathic TN. A common denominator in the clinical phenomenology, initially described already as early as in the 2th century CE (see Nurmikko and Eldridge^2^) is the paroxysmal pain component, usually only a few seconds in duration, not infrequently accompanied by a “tic” (i.e. a brief facial twitch movement) on the affected side. In addition, some patients report an “atypical” concomitant continuous or near-continuous ongoing pain on which the paroxysms are superimposed. These sufferers always have an initial history of paroxysmal pain only. The majority of patients also report trigger zones, extra- or intraorally, where mild tactile stimuli may induce a painful tic. Not infrequently a period of refractoriness will be evident so that new triggers fail to induce a pain paroxysm for a short period of time. If, for example, chewing, intraoral stimuli or swallowing trigger an attack, the refractory period can be used to literally shove food down the gastrointestinal tract, before sensitivity returns.

Explorations in animal models are used for the understanding of disease mechanisms potentially translatable into development of new therapies for patients. In neurobiology, the degree to which these models are successful in mimicking the disease that is investigated varies, and is frequently not satisfactory (e.g. see literature^3–5^). Based on these considerations we conclude that from a scientific as well as an ethical standpoint, animal experiments in pain research should be subjected to rigorous experimental design. Keeping this in mind, it is conspicuously disturbing to note that according to Pub Med, between 2016 and 2019 at least 21 articles have been published where the authors claim that they are using an animal model of TN (e.g. see previous works^6–11^). Among those are articles published in Molecular Pain.

These reports, performed on a rodent model that is supposed to reflect TN in humans, are to us a long overdue wakeup call that must not pass unnoticed. The model used is a mechanical injury to the infraorbital nerve, the major branch of the n.V:2, which is said to induce the specific painful symptoms and signs of TN in all animals included. It is far from clear to the reader why TN is brought forward in these studies, since the same injury in other work is postulated to induce a painful trigeminal neuropathy (for references, see e.g. Ding et al.^12^), a condition significantly different from TN not only from the clinical phenomenology perspective,^13^ but also regarding evidence-based treatment.^14^

The molecular mechanisms behind TN are still unknown, and the quest for it constitutes a major challenge for neurobiology and neurology. Some 25 years ago, Devor and colleagues based on electrophysiological data and clinical observations suggested that the mechanism behind TN characteristics in some specific etiologies such as triggering, amplification and discontinuation of pain attacks is related to altered (increased) excitability of trigeminal ganglion neurons (the ignition hypothesis, see Rappaport and Devor^15^ and Devor et al.^16^). This hyperexcitability would be a sequel of nerve injury and demyelination, coming from e.g. compression of the trigeminal nerve root by a blood vessel close to the brainstem. Accordingly, the situation is improved in some, but not all, patients with this etiology who undergo surgical intervention to decompress a nerve root. Some patients with various etiologies of TN also benefit from glycerol injection, thermocoagulation or balloon compression of the trigeminal ganglion, alternatively from gamma irradiation in the trigeminal root entry zone.^17^

Experimental damage to the infraorbital nerve as a model for TN is indeed questionable. When this nerve was injured in the rat using cobra venom, it was claimed that significant changes in exploratory, resting, face-grooming, and head-shake behaviors appeared after surgery.^18^ These alterations, which were ascribed to demyelination in the infraorbital nerve, do not mimic the clinical features of human TN as noted above. A couple of studies have actually followed the lead regarding mechanical pressure on the trigeminal root as cause for TN.^19,20^ In one, crystals of a superabsorbent polymer were placed next to the trigeminal nerve root of rats, thereby causing ongoing compression of the trigeminal nerve root.^20^ However, the subsequent testing demonstrated only increased sensitivity to mechanical stimuli in the face but spontaneous paroxysmal attacks (i.e. the hallmark of TN) were not reported. Thus, it was not a model of TN, although this was stated even in the title of the paper.

In conclusion, we urge researchers in the field, and certainly referees of data that is submitted for review, not to take TN lightly. As far as we know, there is no animal model that even remotely meet the criteria for a TN model. TN is a devastating disease for those patients who do not get relief from evidence-based interventions, such as administration of carbamazepine/oxcarbazepine,^17^ or from neurosurgical strategies such as those mentioned above. The remaining sufferers need serious innovative attempts in neuroscience and neurology to receive help.
